# Clearing and Masking Agents in Pretargeting Strategies

**DOI:** 10.3390/ph16040497

**Published:** 2023-03-27

**Authors:** Markus Staudt, Matthias M. Herth

**Affiliations:** 1 Department of Drug Design and Pharmacology, Faculty of Health and Medical Sciences, University of Copenhagen, Jagtvej 160, 2100 Copenhagen, Denmark; 2 Department of Clinical Physiology, Nuclear Medicine & PET, Rigshospitalet, Blegdamsvej 9, 2100 Copenhagen, Denmark

**Keywords:** tetrazine ligation, pretargeting, bioorthogonal, clearing agents, masking agents

## Abstract

‘Pretargeting’ led to increased target-to-background ratios of nanomedicines in short timeframes. However, clearing or masking agents are needed to reach the full potential of pretargeted approaches. This review gives an overview of clearing and masking agents employed in pretargeting strategies in both preclinical and clinical settings and discusses how these agents work.

## 1. Introduction

### 1.1. Pretargeting in Nuclear Imaging and Radionuclide Therapy

The recent development of pretargeted radioimmunoimaging strategies has alleviated one of the limitations of conventional, directly radiolabeled monoclonal antibodies (mAb). Pretargeting strategies separate the injection of the mAb and the radioisotope effector. This drastically reduces imaging timeframes from 2–3 days to a few hours, therefore decreasing off-target exposure and significantly reducing radiation burden for patients. The use of a clearing or masking step before injection of the radioisotope effector can facilitate the clearance or blocking of unbound mAbs in the blood. As these strategies can further increase the target-to-background ratios by a factor of up to 125-fold, it is of utmost interest to integrate clearing or masking strategies into pretargeted approaches. The high interest and rapid progress in pretargeted radioimmunoimaging and -therapy is reflected in a large number of reviews discussing different aspects of pretargeting (for example, comparison to other radionuclide delivery strategies [1], different bioorthogonal vectors [2,3,4,5], clinical trials [6], as well as its therapeutic or theranostic applications [7,8,9,10,11]). Yet, no review has focused on the aspects and implications of different clearing and masking strategies. As these are considered an integral step towards clinical translation, this review will focus on agents employed in pretargeted imaging and therapy to facilitate the excretion or masking of unbound mAbs in the blood.

### 1.2. Bioorthogonal Strategies

In pretargeting, there are multiple ways to target pre-accumulated nanomedicines, i.e., mAbs, nanobodies, polymers, proteins, or other targeting vectors. In general, both covalent and non-covalent strategies exist [12]. The latter utilizes high-affinity interactions between, e.g., bispecific antibodies recognizing the target antigen as well as a hapten of choice or the hybridization of complementary deoxyribonucleic acids (DNA), peptide nucleic acids (PNAs), or phosphorodiamidate morpholino oligomers (MORFs). While a few examples of these systems have been reported, the most commonly utilized system is based on the interaction between biotin and (strept)avidin. Several clinical studies have been initiated and are ongoing. Within these studies, clearing strategies have been developed and are often employed.

While non-covalent pretargeting was among the earliest reported strategies, its use has seen a decline, while covalent bond formations via bioorthogonal chemistry have seen rapid growth. This is mostly due to reduced immunogenic response as well as more straightforward production of the required conjugates. While there are a few reports on pretargeting with strain-promoted azide-alkyne click (SPAAC), the relatively slow kinetics of around 1.0 M^−1^s^−1^ severely limits its use in vivo. In contrast, the much faster kinetics of the inverse electron-demand Diels-Alder (IEDDA) reaction between tetrazine (Tz) and trans-cyclooctene (TCO) ligation shows excellent speed kinetics (10^6^–10^7^ M^−1^s^−1^) and bioorthogonality. This has led to a rapid rise in this ligation for pretargeted strategies and in-depth investigations on the required speed kinetics, [13] biodistribution of employed tetrazines [14], and various radiolabeling techniques for radiometals [15,16], as well as direct and indirect labeling with short-lived radionuclides such as carbon-11 (^11^C) or fluorine-18 (^18^F) [17,18,19,20].

### 1.3. Clearing Versus Masking—What Are the Basics behind These Strategies?

**Figure 1:**
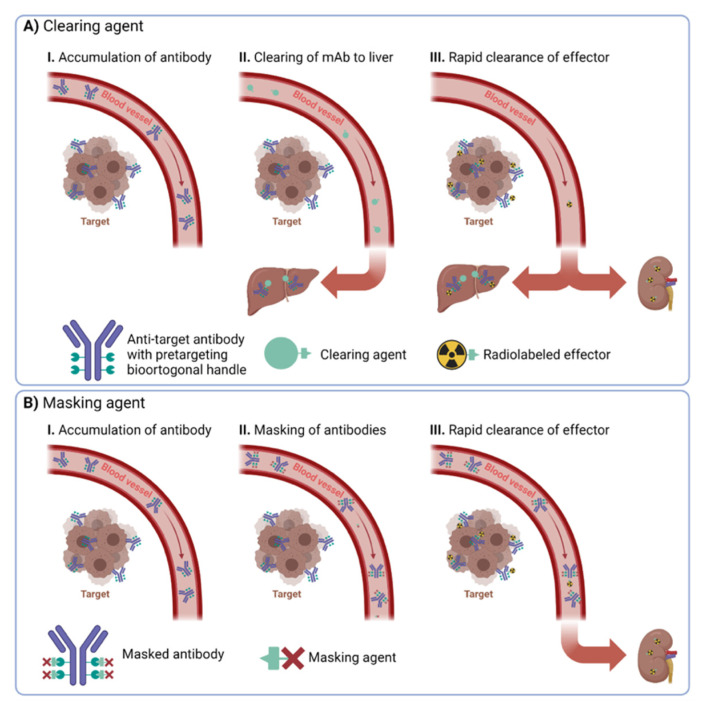
Overview of the difference between (**A**) clearing and (**B**) masking agents.

Clearing agents (CA) guide nanomedicines from the bloodstream into a specific organ, often an excreting organ such as the liver or the kidney (Figure 1A). Typically, the clearing agent is conjugated in vivo to the nanomedicine through either covalent or non-covalent interactions with pre-installed bioorthogonal handles. In this approach, usually, not all handles have reacted with clearing agents. Therefore, radioisotope effectors can still ligate to the nanomedicine when it has been guided to the excretion organ. Consequently, high uptake of the radioisotope effector can often be observed in the respective excretion organ. This might limit the application of the approach as maximum tolerated dose levels might be exceeded relatively quickly in the excreting organ.

Masking agents make use of a different approach. They aim to mask the bioorthogonal handles of the nanomedicine solely in the blood. Consequently, radioisotope effectors cannot ligate to them in the blood. In contrast to clearing approaches, masking agents need to ligate to almost all bioorthogonal handles of the nanomedicine in the bloodstream (Figure 1B). Especially challenging in any masking agent approach is to design the agents in such a way that they cannot interact with the bioorthogonal handles of the nanomedicine at the target site. This is much more important for masking agents compared to CA strategies, as almost all handles should be occupied by circulating nanomedicine, as otherwise, they lose their effect. This is usually achieved by designing masking agents that cannot penetrate easily into the tumor vasculature. When successful, masking agents bear the advantage that radioisotope effectors should find only bioorthogonal handles in the target region and not in any off-target tissues.

## 2. Clearing Strategies

### 2.1. Carbohydrate-Based Clearing Agents

The most often utilized clearing system is based on dendrimeric or polymeric structures bearing sugar moieties such as dextrans or *N*-acetylgalactosamines (GalNAc). Dextrans are branched polysaccharides consisting of many glucose molecules tethered through α-1,4 or α-1,6 linkages, forming chains of varying lengths. Clearing agents based on polymeric dextrans typically have molecular weights of ~200–500 kDa. These large structures bind to the circulating nanomedicine in the blood and induce excretion via the liver. The excretion proceeds through recognition and catabolism by the reticuloendothelial system [21,22,23]. Intratumoral extravasation of these particles and blocking of reactive handles of the pre-accumulated pretargeting vector within the tumor is minimized simply by the size of the clearing agents. Structures in the size of ~200–500 kDa do not accumulate within the tumor—for example, via the EPR effect—in timeframes in which dextrans are already excreted [24,25]. Opposed to the polydisperse nature of dextrans leading to potential reproducibility issues, GalNAc-based CAs are well-defined dendrimeric structures bearing up to 32 GalNAc units. Their synthesis is well described, and well-defined loadings can be accessed with minimal synthetic effort [26]. Excretion is achieved through the Ashwell-Morell receptor, a lectin highly expressed on the surface of mammalian hepatocytes in the liver [27,28]. These receptors recognize terminal galactose and GalNAc residues and remove them from circulation [29].

#### 2.1.1. Efficiency of Clearing

The efficiency of different carbohydrate-based CAs is listed in Table 1. In general, the tumor-to-blood ratio (TBR) can be increased by a factor between 6 and 48 using Cas. For example, the Larson group studied the effect of dextran and GalNAc_16_-based clearing agents on the biodistribution of an anti-GPA33/anti-DOTA (tetraazacyclododecane-tetraacetic acid) bsAb huA33-C825 construct. Both clearing agents showed similar clearing efficiencies, with a 26-fold increase in tumor-to-blood ratios (Table 1, entry 1). The high efficiency of dextran clearing agents in DOTA-hapten-based pretargeting was confirmed in three further studies, employing bsAbs of scFv C825 bound to hu3F8 (anti-GD2), huA33, and trastuzumab (anti-HER2), respectively (Table 1, entries 2–4). In these cases, increased TBR of 21-, 68-, and 7-fold were found.

In a study based on the biotin/streptavidin pretargeting pair—using a CC49 scFv_4_-streptavidin fusion protein (scFv_4_-SA)—the quantity of GalNAc units per clearing agent was investigated with respect to their clearing abilities (Table 1, entry 5) and compared to those of dextran. The clearing agent bearing 32 GalNAcs showed the highest TBR of 639.8, i.e., a 21-fold increase over the control without CA was observed. In comparison, the dextran-based CA showed only an 8-fold increase and was outperformed by nearly all GalNAc systems in this study.

Another study by the Larson group employed anti-GD2 scFv 5F11 bound to streptavidin (5F11-SA) and ^111^In-labeld DOTA-biotin (Table 1, entry 7). Different doses of GalNAc_16_ CA ranging from 15 to 900 µg were investigated, leading to TBR spanning from 71.8 using 15 µg CA to a TBR of 1040 using 450 µg CA. Surprisingly, a 900 µg CA dose did not show any further increase, indicating a plateau of the clearing effect at high doses.

#### 2.1.2. Preclinical and Clinical Translation

Given the high clearing efficiencies of these carbohydrate-based CA, a large number of preclinical and clinical studies were initiated. A short, non-comprehensive overview of CA, mAb, pretargeting handle, and radionuclide used in these studies can be found in Table 2.

In these studies, typically, the biodistribution or TBRs of the directly radiolabeled mAb was compared to the pretargeted approach with and without CA. Usually, CA was administered using a dose escalation regime to identify the optimal CA dose. Identifying the optimal CA dose is another parameter that increases the complexity of the pretargeted approach and does not make its applicability easier. Only drastically increased TBRs can justify discussed CA approaches. However, observed TBRs are in such order that it appears that the benefit of the approach indeed outweighs the increased complexity (see Table 1, for example). In fact, the aforementioned studies highlight the superiority of pretargeted strategies over directly labeled antibodies in terms of tumor regression and progression-free survival.

**Figure 2:**
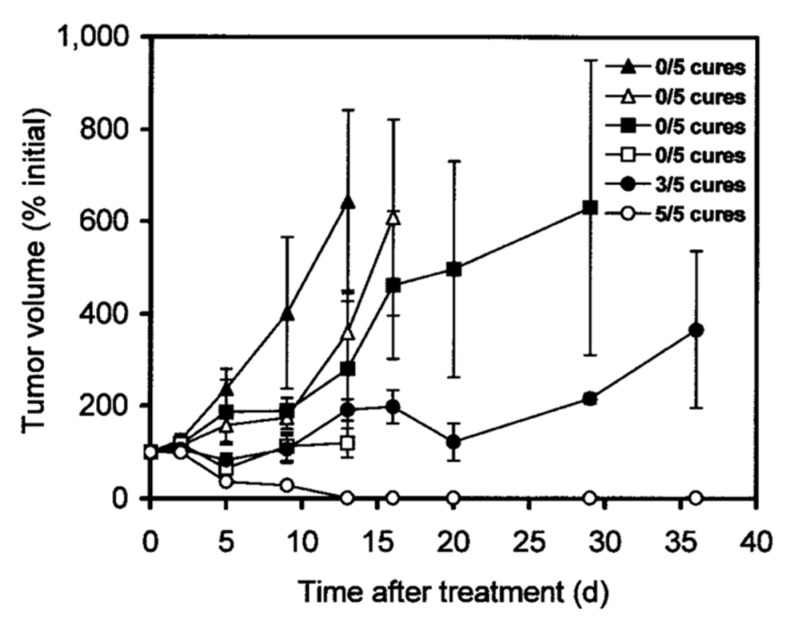
Treatment study comparing pretargeted and directly labeled radioimmunotherapy. Ramos lymphoma xenograft-bearing mice were injected with saline alone (▲), with control NR-LU-10-sAv followed by CA and later by 29.6 MBq (800 µCi) of ^90^Y-DOTA-biotin (△), with 7.4 MBq (200 µCi) (■) or 14.8 MBq (400 µCi) (□) of directly labeled ^90^Y-1F5, or with 1F5-sAv followed by CA and later by 14.8 MBq (400 µCi) (●) or 29.6 MBq (800 µCi) (○) of ^90^Y-DOTA-biotin. Image from Ref. [39].

For example, the group of Press evaluated the mAb 1F5 targeting CD20 overexpressed in Ramos lymphoma xenografts (Table 2, entry 3) [39]. The mice were injected with 1F5-streptavidin, followed by GalNAc_16_-biotin and 14.8 MBq or 29.6 MBq of ^90^Y-DOTA-biotin. Another cohort received 7.4 MBq or 14.8 MBq of directly labeled ^90^Y-DOTA-1F5 (Figure 2). The conventionally labeled ^90^Y-DOTA-1F5 eventually showed progressive tumor growth and no complete remissions. In contrast, the pretargeted group showed a high decrease in tumor volume as well as 60% and 100% complete tumor remission for the low and high doses, respectively. Furthermore, no significant weight loss or signs of toxicity were observed in the pretargeted approach. This shows that—using the pretargeted approach with CAs—higher radiation doses are tolerated, and therefore increased therapeutic effects can be achieved.

Based on these promising results, clinical trials were initiated to take advantage of the higher tolerated radiation doses realized through pretargeting with CAs. A study by Forero et al. using 15 mCi/m^2^ (555 MBq/m^2^) ^90^Y-DOTA-biotin reported complete remissions in two and partial response in 1 out of 15 cases of B-cell non-Hodgkin lymphoma (Table 2, entry 12). No significant hematologic toxicity was observed in 12 patients.

A treatment study targeting the same cancer type (Table 2, entry 13) in 7 patients showed 2 complete remissions, 1 unconfirmed remission, and partial responses with 80% tumor volume reduction and 2 patients with approximately 50% tumor volume reduction. Only one patient showed progressive disease. Both doses of 30 and 50 mCi/m^2^ (1.11 and 1.85 GBq/m^2^) ^90^Y-DOTA-biotin showed no significant toxicity.

These results indicate that the pretargeting concept, in combination with GalNAc-based CA, can be translated into humans, allowing for higher radiation doses than possible with traditional directly labeled mAbs.

### 2.2. Serum Albumin-Based Clearing Agents

Human serum albumin (HSA) is the most abundant protein in human blood plasma. Its long plasma half-life of 3 weeks makes it an unsuitable clearing agent on its own. To facilitate clearing to the liver, HSA has to be conjugated into galactose units; usually, 40 units are required. This modification results in a rapid excretion via the liver induced by the binding of these galactose units to the Ashwell–Morell receptor [29].

#### 2.2.1. Efficiency of Clearing

**Figure 3:**
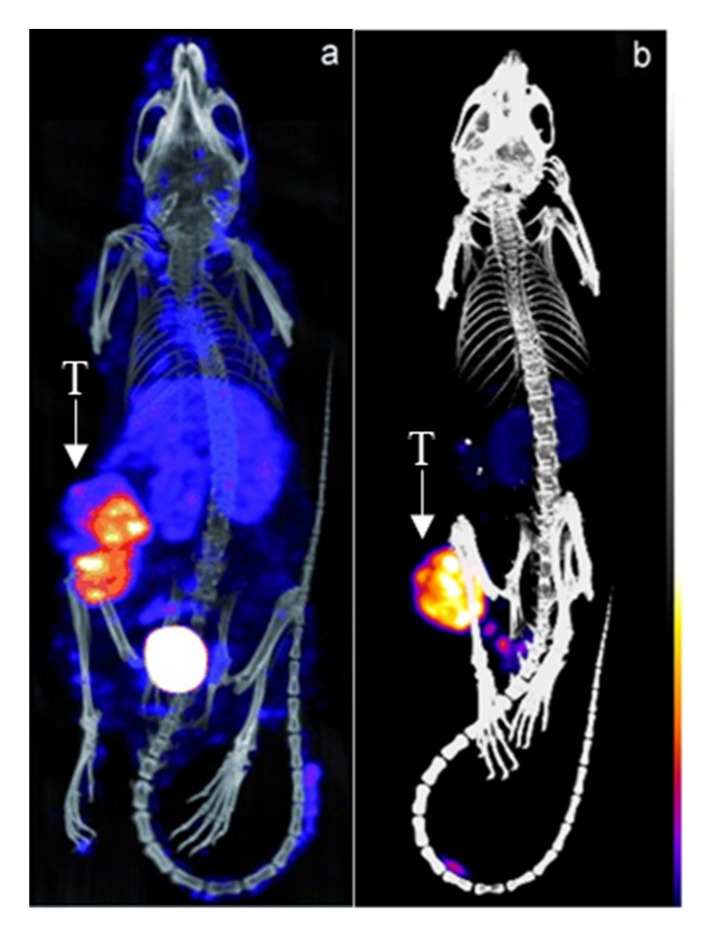
Pretargeted imaging of LS174T-tumor bearing mice using CC49-TCO and ^111^In-DOTA-tetrazine (**a**) without and (**b**) with an albumin-based CA. T = tumor. Images from Refs. [53,54].

A pretargeting study by Rossin et al. using the TCO-conjugated anti-TAG72 mAb CC49 showed a 125-fold improvement of TBR and doubled the uptake of the ^111^In-DOTA-tetrazine upon utilization of an albumin-based CA (Figure 3) [53]. The 2 evaluated CAs were mouse serum albumin conjugated with 15–19 galactose and 9–13 bispyridyl-Tz units and 0.5 µm polystyrene beads coated with bovine serum albumin (BSA) bearing 8–10 bispyridyl-Tz units. While both showed efficient blood-clearing of ^125^I-labeled CC49-TCO within 30 min of 22- and 12-fold, respectively, the polystyrene-based CA showed an increase in blood radioactivity after the initial drop and higher liver and spleen uptake. Further dosing optimization of the galactose-BSA-Tz CA in pretargeting experiments using ^177^Lu-DOTA-Tz revealed that 160 μg CA administered 30 h post-CC49-TCO injection increased TBR to 46. Using 2 cycles of CA at 30 and 48 h increased contrast even further to 254-fold. Interestingly, the 2-dosing regimen had no major influence on tumor uptake (7.45 ± 1.46% ID/g vs. 6.13 ± 1.09% ID/g with 1 and 2 CA doses), indicating minimal intratumoral extravasation of the galactose-BSA-Tz.

**Figure 4:**
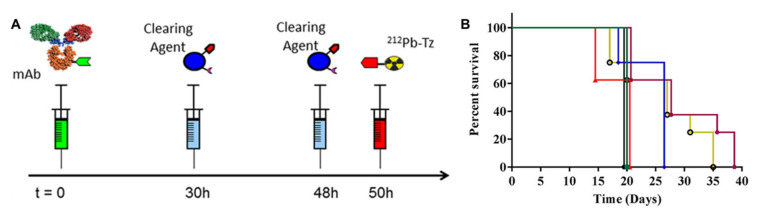
**(A**) Schematic representation of the pretargeting protocol used. mAb CC49-TCO, CA galactose-BSA-Tz, and ^212^Pb-DOTA-Tz were used. (**B**) Kaplan–Meier survival curves. LS174T tumor-bearing mice were treated with CC49-TCO mAb and treated with CC49-TCO (green ■), 2.78 MBq (blue ⧫), 4.63 MBq (yellow ○), 7.40 MBq (red ▲), and 2 × 2.78 MBq (purple ⬣) of ^212^Pb-DOTA-Tz. Reprinted with permission from Ref. [56]. Copyright 2017, American Chemical Society.

The high clearing efficiency of this system was confirmed in a later study employing a slightly modified CC49-TCO, leading to a TBR of 304 using 2 doses of CA [55]. Furthermore, the same dosing regimen and CA were also utilized for pretargeted α-therapy using ^212^Pb-DOTA-Tz [56]. The pretargeting protocol allowed for 5–10 times higher dosing than the directly labeled approach and led to statistically significant increased survival over the vehicle control (Figure 4). A dose-dependent reduction in tumor size could be observed, although mice in the highest dose (7.40 MBq) had to be sacrificed on day 20 due to hematological radiation toxicity and poor body score index.

#### 2.2.2. Preclinical and Clinical Translation

In a clinical optimization study by Breitz et al., the influence of different dosages of a CA on safety, biodistribution, and antiglobulin formation was investigated [57]. The pretargeting was based on a ^90^Y-DOTA-biotin and streptavidin-conjugated IgG mAb NR-LU-10, targeting the epithelial cell adhesion molecule (EpCAM) overexpressed in epithelial tumors. The CA was based on an HSA bearing 2 biotin and 40 galactose units. The study identified the optimal timing interval of 48 h between injection of mAb and CA and 24 h between CA and radiolabeled biotin. Furthermore, no difference between CA injections of two doses at varying time points over 24 h, intravenous bolus, or infusion over a 24 h period was found. The ideal molar ratio between the mAb-conjugate and CA was found to be 1:10, with higher doses leading to a reduced uptake of radiolabeled biotin. As frequently observed when applying pretargeted systems based on the biotin/streptavidin pair, an immune response to this treatment was detected. The formed immunogenic antibodies against streptavidin and the mAb-conjugate did not disturb the clearing abilities of the CA. However, this immunogenicity is a potential impediment, especially considering subsequent injections. It will lead to reduced bioavailability and altered pharmacokinetic properties, ultimately leading to reduced efficacy of the administered dose.

Using the aforementioned optimized protocol, a phase II study was conducted by Knox et al. in 25 patients receiving a dose of 110 mCi/m^2^ (4.07 GBq/m^2^) ^90^Y-DOTA-biotin [58]. The overall therapeutic efficacy was quite low, achieving an overall response rate of 8%. Hematological and nonhematological toxicity were observed. The overall poor outcome of the study was attributed to off-target toxicity due to the binding of the NR-LU-10 mAb to collecting tubules in the kidney and gastrointestinal epithelium. Still, the study could show successful proof-of-concept of the clearance approach.

### 2.3. Other

#### 2.3.1. Monoclonal Antibody-Based Clearing Agents

Myrhammar et al. investigated the validity of lactosaminated mAb-based clearing agents in a PNA pretargeted system [59]. For this, trastuzumab was conjugated to PNA and labeled with iodine-131. Six hours later, lactosaminated cetuximab conjugated to the complimentary PNA was injected, and the mice were sacrificed one hour later. Only a minor reduction in blood radioactivity levels from 8.5 ± 1.8 to 6.0 ± 0.4% ID/g was observed. This was mostly attributed to the low conjugation yields of the complementary PNA to the CA of approximately 45%.

In a pretargeting study by Karacay et al. and Sharkey et al., MN-14, an anti-carcinoembryonic antigen (CEA), mAb, was conjugated with streptavidin [60,61]. The CA was WI2, an anti-antibody against MN-14, which was galactosylated to induce excretion via the liver. It was found that more than 14 galactose units were required to achieve this, with 44 giving the best clearing effect. Using a 5-molar excess of the latter gave an approximately 20-fold decrease in ^111^In-DTPA-peptide-biotin blood levels 24 h after injection of the CA.

In a follow-up study, the same CA was found to be efficient in clearing bsAb MN-14 conjugated c734 (MN-14 × c734), an anti-(In) DTPA Fab’ [62]. The ^99m^Tc-labeled peptide IMP-192 bearing two (In) DTPA moieties was used for evaluation. The blood levels of the peptide were reduced from 11.3 ± 2.9% ID/g to 0.9 ± 0.28% ID/g; therefore, the TBR was increased from 1.84 ± 0.87 to 11.3 ± 2.9. Furthermore, the influence of galactosylation of the mAb on the clearing effect was investigated. At both time points, 5 and 24 h after CA injection, TBR for gal-WI2 was found to be superior to the native mAb WI2 (3.07 ± 2.02 vs. 10.4 ± 11.2 and 9.25 ± 9.92 vs. 19.0 ± 18.8, respectively).

#### 2.3.2. Avidin-Based Clearing Agents

Avidin as a chase/CA was used by Paganelli in a so-called three-step pretargeting procedure and was among the first utilizations of CA to increase TBR [63,64,65]. The three steps typically consist of (1) injection of a biotinylated mAb; (2) after maximal target accumulation, CA avidin administration; and (3) imaging through a biotinylated radioisotope effector.

Given avidins’ tetrameric structure, it causes aggregation of biotinylated mAbs in the bloodstream and therefore excretion through the reticuloendothelial system as well as removing endogenous biotin from the bloodstream [66]. Apart from that, binding to the pre-accumulated biotinylated mAb causes an increase in potential binding sites for the biotin-radioisotope effector. This system gave increased TBR of up to 10-fold while not impacting tumor accumulation [67,68].

Unfortunately, due to the nature of the clearing mechanism, it cannot be translated to more promising covalent strategies such as TCO-tetrazine ligation. Given the antigenicity caused by the (strept)avidin and the concomitant transition to less immunogenic pretargeting pairs, it makes further utilization of this CA challenging. This might explain the rapid decline in pretargeting studies utilizing avidin CA in the past decade.

#### 2.3.3. Apotransferrin-Based Clearing Agents

Apotransferrin (aTf) is a slowly diffusible serum protein, which can be conjugated to an antigen and thus be utilized as a clearing agent. Excretion proceeds through the formation of cross-links between antibodies, leading to rapid removal through the reticuloendothelial system, similar to previously discussed dextrans.

A study by Goodwin et al. utilized mAb WC3A11 binding strongly to the chelator DOTA. As the mAb lacks any tumor-targeting bispecificity, the accumulation occurred nonspecifically through leaky neovasculature in the tumor. The aTf-DOTA clearing agent showed an increase in TBR from 1.80 to 8.72, although tumor uptake was lowered at the same time from 4.07 ± 0.33% ID/g to 1.40 ± 0.61% ID/g (Table 3, entry 1). This indicates the CA-blocking binding sites of the pre-accumulated mAb in the tumor. In a follow-up study, replacing the monovalent hapten with a bivalent one, the tumor uptake could be substantially improved to 7.4 ± 1% ID/g [69].

The same CA was also used in combination with the mAb to 2D12.5, strongly binding to yttrium(III)-labeled DOTA. After nonspecific accumulation in the KHJJ tumors, the CA was injected, followed by either mono- or bivalent ^88^Y-DOTA after 20 h. The bivalent radioisotope effector achieved a superior TBR of 21 compared to 16 for the monovalent ^88^Y-DOTA [72]. This can be attributed to the higher observed tumor uptake of 4.41 ± 1.63% ID/g compared to 1.74 ± 0.83% ID/g.

A study by Schuhmacher et al. employed the chelator *N*,*N*′-Di(2-hydroxybenzyl)ethylene-diamine-*N*,*N*′-diacetic acid (HBED) and the corresponding clearing agent aTf-HBED (Table 3, entry 2). bsAb targeting the glycoprotein CD44v and Ga-HBED was injected into 14ASML-1 tumor-bearing mice, followed by aTf-HBED after 24 h and ^67^Ga-HBED 15 min later. A dose-dependent clearing effect was observed, with the highest dose (0.1 nmol/per mouse (23.7 ± 1.8 g)) giving the best result with a 17-fold increase in TBR over control. The same CA was later utilized with an anti-MUC1/anti-Ga-HBED bsAb, resulting in a comparably high TBR of 2.6 [73].

## 3. Masking Strategies

Only a few reports of the utilization of blocking or masking agents in pretargeting have been reported to date. In an extensive study by Karacay et al., the effect of masking agents was compared to the previously mentioned mAb-based clearing of bsAb MN-14 × c734 through the anti-antibody WI2 (see Section 2.3.1 for details) [62]. This was achieved through blocking the ^99m^Tc-IMP-192 binding site of anti-DTPA Fab’ c734 using either non-radioactive indium-loaded IMP-192, an IgG conjugated to 4 and 8 units of DTPA or galacytosylated as well as non-galactosylated BSA conjugated to 4.4 or 8.3 DTPA. Evaluation through injection of ^99m^Tc-IMP-192 was completed at several time points. Furthermore, ^125^I-labeling of the bsAb was performed to monitor blood levels of the masked antibody. The dose of the masking agent was based on the blood levels of ^125^I-bsAb at the time point of injection. Despite increased TBR after employing the masking agents, no significant change in the blood levels of the ^125^I-bsAb was observed, confirming that the increased contrast stems from a masking rather than clearing effect. The results are summarized in Table 4. In-IMP-156 and galactosylated BSA-DTPA were found to only marginally increase TBR by 2.5 to 5-fold (Table 4, entry 2–4). In contrast, 5 equivalents of BSA-DTPA in both DTPA-loadings had an increased TBR of 55–60-fold (Table 4, entry 6, 7). The optimal time point for injection of ^99m^Tc-IMP-192 was found to be 2 h, with shorter and longer masking times leading to drastically reduced contrast (Table 4, entries 8–11). Surprisingly, an IgG conjugated to DTPA seemed to be less impactful on the masking time, with both 30 min and 4 h showing high TBR of 9.7 ± 8.3 and 15.3 ± 6.9, respectively (Table 4, entry 12, 13). Overall, this study showed that masking agents based on large molecular conjugates to serum albumins or IgGs could efficiently block binding sites on mAb in the bloodstream without negatively affecting tumor accumulation of the radioisotope effector. The achieved TBR was similar to the CA based on secondary anti-antibodies, but the liver uptake was drastically reduced by 5 to 15-fold due to the masking effect. Considering the more straightforward production and broad commercial availability of serum albumins, this study highlights the benefits of masking over clearing agents.

In a study by the groups of Lewis and Reiner, the masking concept was applied to pretargeting using the TCO-tetrazine ligation [74]. For this, tumor-bearing mice were injected with TCO-conjugated mAb huA33, targeting the A33 antigen, or mAb 5B1-TCO, targeting carbohydrate antigen 19-9. After 48 h post-administration, a masking agent was injected—a 2000 kDa dextran polymer decorated with approximately 60–600 tetrazine moieties. It was found that 10 min of masking time was sufficient to achieve full ligation of all TCO-mAb constructs in the bloodstream. Following injection of a gallium-68 labeled tetrazine, an increase in TBR from 0.7 ± 0.2 and 0.8 ± 0.2 in the control cohort to 5.8 ± 2.3 and 3.2 ± 0.5 at 2 h post-injection was observed for 5B1-TCO and huA33-TCO, respectively. No significant differences between masking times of 10 to 120 min were found. Furthermore, no significant change in tumor uptake was observed, indicating minimal leakage of the masking agent into the tumor vasculature.

The same masking agent was also evaluated using lutetium-177 labeled Lu-DOTA-tetrazine, and the mAb construct huA33-TCO (3 TCO/mAb), a huA33-DEN-TCO (8 TCO/mAb) analog using a dendrimeric site-specific method rather than direct conjugation method [75]. This mAb conjugate was shown to increase tumor uptake ~2-fold upon long accumulation times of 120 h compared to the dendrimer-lacking huA33-TCO. However, this approach led to increased blood uptake from 0.7 ± 0.2% ID/g (huA33-TCO) to 1.9 ± 0.4% ID/g (huA33-DEN-TCO). While the dextran-tetrazine masking agent managed to decrease blood uptake by 0.3 ± 0.1% ID/g, tumor uptake was also significantly lowered from 23.0 ± 2.2 to 7.5 ± 1.9% ID/g. Furthermore, the masking agent led to a 14-fold increase in lung uptake, which has not been observed for the dendrimer-lacking mAb. This could be explained by the higher TCO load on huA33-DEN-TCO leading to the formation of large particles through cross-linking of individual dextran polymers. These high-molecular-weight particles are often reported to accumulate within the lungs [76,77]. Therefore, this masking agent does not seem suitable for this particular dendritic mAb-construct, and further investigation to reduce the lung uptake as well as extensive optimization is needed to achieve better TBR.

## 4. Conclusions and Outlook

Several CA strategies have been developed over the years since pretargeting was deployed in the late 1980s. So far, avidin-, carbohydrate- and albumin-based CA have been applied in the clinic. Most of these CAs were based on the (strept)avidin-biotin interaction and caused immunogenic responses, leading to a rapid decline in the utilization of those CA. More modern pretargeting strategies, such as TCO-tetrazine ligation, are slowly starting to incorporate CA to further increase TBR. Still, more (pre)clinical evaluations and comparison studies need to be performed to confirm the translatability of these agents.

In contrast, very few masking agents are reported despite promising results being disclosed. Given their inherent benefit of rendering the blood-located nanomedicines invisible to the injected radioisotope effector, they might be a highly valuable addition to the pretargeting tool kit. Still, the generality of the approach and the effectiveness compared to the more established CA needs to be confirmed. So far, there is a distinct lack of investigations utilizing clearing or masking agents and fluorine-18-based radioisotope effectors. Given the ideal nuclear properties of ^18^F for PET with higher resolution and reduced radiation burden, more studies need to be performed to confirm that higher TBR can be obtained. This would be especially beneficial considering further translation towards theranostic approaches using the fluorine-18/astatine-211 pair.

Furthermore, the increased complexity of both clearing and masking agents is still of concern. Not only does the use of additional components increase parameters in need of optimization, but it also makes clinical approval more demanding due to the multitude of possible conjugates formed in vivo. This is especially a concern for the polydisperse nature of some of the agents discussed in this review. Still, the use of these agents represents a large improvement in pretargeting technology and outweighs the added complexity, as evidenced by the successful clinical translation into humans.

In summary, there is still extensive work to be performed to find the ideal clearing or masking setup while trying to minimize an increase in the complexity of the pretargeting system. Systems that are more efficient could vastly accelerate the translation of very promising results seen for IEDDA-based pretargeted immunoimaging aimed at therapeutic radionuclides.

## Figures and Tables

**Table 1:** Overview of carbohydrate-based clearing agents.

Entry	Clearing Agent	Dose of CA	Pretargeting Pair	Radio-Nuclide	Blood (% ID/g) w/o CA w/CA		Tumor-to-Blood Ratio w/o CA w/CA		Ref.
1	Dextran	62.5 µg, 0.125 nmol	bsAb huA33-C825, Hapten	^177^Lu	11.9 ± 0.36	0.45 ± 0.09	**2.9 ± 0.4**	**77.3 ± 19.2**	[30]
	GalNAc_16_	20 µg, 2.2 nmol			11.9 ± 0.36	0.46 ± 0.13	**2.9 ± 0.4**	**59.2 ± 20.0**	
	GalNAc_16_	25 µg, 2.8 nmol			11.9 ± 0.36	0.40 ± 0.18	**2.9 ± 0.4**	**76.4 ± 36.2**	
2	Dextran	250.0 µg, 0.49 nmol	bsAb hu3F8-C825, Hapten	^177^Lu	3.8 ± 0.12	0.14 ± 0.02	**3.5 ± 0.4**	**73.5 ± 10.5**	[31]
3	Dextran	62.5 µg, 0.125 nmol	bsAb huA33-C825, Hapten	^177^Lu	~8	~0.1	**2.2 ± 0.4**	**105.8 ± 52.3**	[32]
4	Dextran	62.5 µg, 0.125 nmol	bsAb trastuzumab-C825, Hapten	^177^Lu	4.95 ± 1.17	~0.3	**4.0 ± 1.2**	**26.7 ± 9.0**	[33]
5	GalNAc_4_	13 µg, 5.7 nmol	scFv_4−_SA DOTA-biotin	^111^In	0.62 ± 0.14	0.02 ± 0.00	**29.8 ± 9.3**	**337.7 ± 103.5**	[34]
	GalNAc_8_	26 µg, 5.9 nmol			0.62 ± 0.14	0.03 ± 0.01	**29.8 ± 9.3**	**174.3 ± 71.7**	
	GalNAc_16_	50 µg, 5.8 nmol			0.62 ± 0.14	0.03 ± 0.01	**29.8 ± 9.3**	**381.2 ± 100.6**	
	GalNAc_32_	100 µg, 5.7 nmol			0.62 ± 0.14	0.02 ± 0.01	**29.8 ± 9.3**	**639.8 ± 317.7**	
	Dextran	100 µg, 0.2 nmol			0.62 ± 0.14	0.02 ± 0.01	**29.8 ± 9.3**	**243.5 ± 85.1**	
6	GalNAc_16_	100 µg, 1.1 nmol	B3-SA DOTA-biotin	^111^In	19.2 ± 1.9	1.71 ± 0.66	**~1.1**	**~7.3**	[35]
7	GalNAc_16_	15 µg, 1.7 nmol	5F11-scFv-SA DOTA-biotin	^111^In	Not reported	~0.11	**Not reported**	**71.8 ± 43.3**	[36]
	GalNAc_16_	75 µg, 8.7 nmol				~0.02		**277.1 ± 74.5**	
	GalNAc_16_	300 µg, 34.7 nmol				~0.02		**263.1 ± 75.2**	
	GalNAc_16_	450 µg, 52.0 nmol				~0.01		**1040 ± 349**	
	GalNAc_16_	900 µg, 140.0 nmol				~0.01		**629 ± 177**	

**Table 2:** Overview of utilized in (pre)clinical studies. In all cases, the CA utilized was GalNAc-based, and the pretargeting pair was a (strept)avidin-mAb in combination with a radiolabeled biotin-DOTA.

Entry	mAb	Target	Therapeutic Radionuclide	Clinical Status	Ref.
1	1F5	CD20	^90^Y	Preclinical	[37]
	HD39	CD22			
	Lym-1	DR			
2	BC8	CD45	^90^Y	Preclinical	[38]
3	1F5	CD20	^90^Y	Preclinical	[39]
4	CC49	TAG72	^149^Pm, ^166^Ho, ^177^Lu	Preclinical	[40]
5	1F5	CD20	^213^Bi	Preclinical	[41]
6	CC49	TAG72	^90^Y, ^177^Lu	Preclinical	[42]
7	B3	Le^γ^	^213^Bi	Preclinical	[43]
8	1F5	CD20	^90^Y	Preclinical	[44]
9	BC8	CD45	^90^Y	Preclinical	[45]
	1F5	CD20			
10	HAT	CD25	^213^Bi	Preclinical	[46]
11	1F5	CD20	^90^Y	Preclinical	[47]
12	B9E9	CD20	^90^Y	Phase I	[48]
13	Rituximab (C2B8)	CD20	^90^Y	Phase I/II	[49,50]
14	CC49	TAG72	^90^Y	Phase I	[51,52]

**Table 3:** Overview of apotransferrin-based clearing agents.

Entry	Clearing Agent	Dose of CA	Pretargeting Pair	Radio-Nuclide	Blood [% ID/g] w/o CA w/CA		Tumor-to-Blood Ratio w/o CA w/CA		Ref.
1	aTf-DOTA	0.75 equiv.	mAb Hapten (DOTA)	^111^In	6.05 ± 0.57	0.16 ± 0.05	1.80	8.72	[70]
2	aTf-HBED	1.7 µg, 0.02 nmol	bsAb Hapten (HBED)	^67^Ga	27.51	17.55 ± 1.04	~0.14	~0.30	
		4.3 µg, 0.05 nmol			27.51	6.12 ± 0.82	~0.14	~1.05	[71]
		8.6 µg, 0.1 nmol			27.51	3.27 ± 0.61	~0.14	~2.43	

**Table 4:** Overview of masking agents used in the study of Karacay et al. Nude mice bearing GW-39 tumors were injected with 150 nmol of bsAb MN-14 × c734, and masking agents were injected 65 h later. Equivalents were based on blood levels of ^125^I-bsAb at that time point. After the shown masking time, ^99m^Tc-IMP-192 was injected, and animals were necropsied 3 h after.

Entry	Masking Agent	Equivalents of Masking Agent	Masking Time (h)	Blood Uptake (% ID/g)	Tumor-to-Blood Ratio
1	None	-	-	20.8 ± 2.9	0.22 ± 0.13
2	In-IMP-156	1.5	0.5	15.7 ± 2.6	0.56 ± 0.19
3	gal-BSA-DTPA4.4	5	2	16.2 ± 2.7	0.35 ± 0.12
4	gal-BSA-DTPA4.4	5	24	14.7 ± 7.7	1.11 ± 1.69
5	BSA-DTPA4.4	1.5	2	5.79 ± 3.19	1.61 ± 1.26
6	BSA-DTPA4.4	5	2	0.90 ± 0.53	12.3 ± 5.2
7	BSA-DTPA8.3	5	2	0.61 ± 0.16	13.4 ± 3.9
8	BSA-DTPA4.4	5	0.5	1.43 ± 0.63	3.55 ± 1.99
9	BSA-DTPA4.4	5	1	1.10 ± 0.60	4.79 ± 1.54
10	BSA-DTPA4.4	5	2	1.00 ± 0.41	14.2 ± 4.9
11	BSA-DTPA4.4	5	24	1.17 ± 0.54	2.20 ± 0.74
12	IgG-DTPA4	5	0.5	1.05 ± 0.72	9.66 ± 8.34
13	IgG-DTPA4	5	4	0.57 ± 0.15	15.3 ± 6.90

## Data Availability

Data sharing not applicable.
